# A ginger extract improves ocular blood flow in rats with endothelin-induced retinal blood flow dysfunction

**DOI:** 10.1038/s41598-023-49598-w

**Published:** 2023-12-20

**Authors:** Nana Takahashi, Kota Sato, Naoki Kiyota, Satoru Tsuda, Namie Murayama, Toru Nakazawa

**Affiliations:** 1 Department of Ophthalmology, Tohoku University Graduate School of Medicine, 1-1, Seiryo, Aoba, Sendai, Miyagi 980-8574 Japan; 2 Department of Advanced Ophthalmic Medicine, Tohoku University Graduate School of Medicine, Sendai, Miyagi Japan; 3 Department of Ophthalmic Imaging and Information Analytics, Tohoku University Graduate School of Medicine, Sendai, Miyagi Japan; 4 Department of Retinal Disease Control, Tohoku University Graduate School of Medicine, Sendai, Miyagi Japan

**Keywords:** Translational research, Therapeutics, Eye diseases

## Abstract

The aim of this study was to investigate the effect of a ginger extract on optic nerve head blood flow (ONH BF) under endothelin-1 (ET-1) stimulation. Using laser speckle flowgraphy, we measured ONH BF in brown Norway rats. To establish the ONH BF impairment profile under ET-1 stimulation, we administered an intravitreal injection of ET-1 under anesthesia. We then gave the ginger extract sublingually to assess its effect on ONH BF in both normal and ET-1-induced ischemic conditions. Post ET-1 injection, there were no significant changes in parameters including intraocular pressure or systemic factors. ONH BF showed a dose-dependent decline after ET-1 injection, with a significant reduction after a 2.50 pmol ET-1 dose. Sublingual administration of the ginger extract significantly improved ONH BF in both normal and ET-1-stimulated rats. This suggests that our newly developed supplement for improving ONH BF has a potential role in retinal ischemic diseases, including glaucoma.

## Introduction

Glaucoma is the second leading cause of blindness worldwide^1,2^. High intraocular pressure (IOP) is the only evidence-based risk factor for glaucoma progression^3^, but glaucoma can progress even with normal IOP. Many non-IOP risk factors have been investigated^4^, but glaucoma pathogenesis remains imperfectly understood. One suspected non-IOP risk factor for progression is damage to the optic nerve head (ONH) and associated low blood flow (BF). Reduced BF leading to insufficiencies in the supply of oxygen and nutrients could potentially contribute to retinal ganglion cell (RGC) loss^5^. Ischemic retinal diseases, such as anterior ischemic optic neuropathy, retinal vein occlusion, and glaucoma, that are accompanied by impaired BF, could contribute to retinal damage, including to the RGCs^6^. Previously, we found that ONH BF impairment (assessed by laser speckle flowgraphy [LSFG] or optical coherence tomography angiography) might be associated with glaucoma severity and progression^7–9^.

Endothelin-1 (ET-1), one of the most potent vasoconstrictor peptides^10^, has been implicated in glaucoma pathogenesis. Several basic research studies have showed that intravitreal injection of ET-1 induces optic neuropathy in rodent models^11–15^. Excessive ET-1 is induced by IOP elevation, retinal ischemia, and inflammatory stimulation, such as with TNFα, resulting in astrocyte activation, in addition to retinal vasoconstriction^11,16,17^. Acute BF reduction in the retina contributes to axonal damage and RGC death, suggesting that ameliorating ET-1-induced vasoconstriction might be a potential therapy to inhibit glaucoma progression.

There are various approaches for improving BF dysfunction, but an ideal approach would be one that is feasible for ingestion as food or a supplement and has demonstrated efficacy. Based on previous reports demonstrating the vasodilatory effects of ginger root and [6]-shogaol on increasing BF^18–21^, this study specifically focuses on the supplement Flow-ginger, which is an extract derived from red ginger (*Zingiber officinale* var. *rubra*). In this study, we investigate the effects of this ginger extract and determine whether it ameliorates ET-1-induced retinal BF reduction.

## Materials and methods

### Animals

Male, 8–12-week-old, pigmented brown Norway rats weighing 190 to 260 g were obtained from Japan SLC (Shizuoka, Japan) and maintained at the Tohoku University Graduate School of Medicine. All rats were kept under controlled light/dark conditions, with food and water available ad libitum. To alleviate animal distress, all procedures, excluding the sublingual administration of ginger extract, were performed under anesthesia. For the anesthesia, a combination of ketamine (100 mg/kg) and xylazine (10 mg/kg) was administered intramuscularly. Euthanasia was carried out using an overdose of sevoflurane inhalation anesthesia. All experimental protocols were approved by the Committee for Animal Experiments at the Tohoku University Graduate School of Medicine (Approval #2022-056) and conducted under the authority of the project license. All rats were handled, and all experiments were performed in accordance with the ARVO Statement Guidelines for the Use of Animals in Ophthalmic and Vision Research and ARRIVE (Animals in Research: Reporting In Vivo Experiments) guidelines.

### Intravitreal injection of ET-1

At first, pupil dilation was performed with 0.4% tropicamide (Mydrin M; Santen Pharmaceutical Co. Ltd., Osaka, Japan). ET-1 (Peptide Institute, Osaka, Japan) was dissolved in phosphate-buffered saline (PBS). Prior to administration, the time elapsed between adjusting the ET-1 dose and administering it was consistently maintained within the same protocol experiment to ensure no variability between the groups. Two microliters of ET-1 (at 1.25, 2.50 or 5.00 pmol/eye) were injected into the vitreous body just above the ONH of the right eye under anesthesia while the ONH was visible under the microscope. Intravitreal injection was achieved by inserting a 33-gauge needle attached to a Hamilton syringe. As a control, an equal volume of PBS intravitreal injection was given to the right eye in the same way. After injection, the rats were kept on a heat pad.

### Measurement of optic nerve head blood flow

We used the LSFG-Micro (Softcare Co. Ltd., Fukutsu, Japan) to evaluate ONH BF. Previously, LSFG-Micro was reported to be a reliable source of information for evaluating circulation in the ONH of rats^22^. LSFG-Micro comprises a charge-coupled device image sensor with a resolution of 600 × 480 pixels and a diode laser with an 830 nm wavelength. These are attached to a stereo microscope (SZ61TR, Olympus Corporation, Tokyo, Japan). The main LSFG variable, expressed in arbitrary units, is termed mean blur rate (MBR) and is an indicator of relative BF.

The device captures 120 continuous fundus images over 4 s; these images are mathematically averaged, producing a composite map of ocular BF. The accompanying analysis software can automatically divide the MBR map into areas characterized by the large vessels and the capillaries of the ONH. Based on this division, values for MBR in the vessels (MV), MBR in the tissue (MT), and overall MBR (MA) were calculated. For our analysis, average MBR was calculated based on three consecutive measurements. We alternated measurements among the rats from each group to minimize measurement bias. We excluded any eyes with corneal opacification or poor image quality. To ensure the integrity of our LSFG image quality, any data with a vascular cloud value below 0.28 was excluded from the study^23^. As shown in Supplementary Figure S1, a circle with a diameter of 1.37 mm was manually placed over the ONH area to identify it, and the vessel region was then automatically segmented by the accompanying analysis software based on a defined MBR threshold. A hydroxyethyl cellulose gel (Scopisol; Senju Pharmaceutical Co. Ltd., Osaka, Japan) was applied to the eyes before a cover glass was placed over them.

### Measurement of IOP and blood pressure

Immediately prior to the LSFG measurements, IOP measurements and heart rate (HR), systolic blood pressure (SBP), diastolic blood pressure (DBP) and mean blood pressure (MBP) measurements were each taken three times. The mean values were calculated and used in the analysis. IOP measurements were taken with a handheld tonometer (Icare; Tiolat Oy, Helsinki, Finland) from each animals’ right eye. An automatic sphygmomanometer (BP-98; Softron, Tokyo, Japan) was used to measure HR, SBP, DBP and MBP in the tail. A formula was then used to calculate mean ocular perfusion pressure (MOPP): MOPP = 2/3 MBP − IOP.

### Experiments on ginger extract powder dosage in normal rats

We conducted an independent experiment to confirm the effectiveness of ginger extract powder at various doses. The ginger extract powder was composed of 50% red ginger [*Zingiber officinale* var. *rubra*] extract and 50% cyclodextrin. The ginger extract powder was provided by Rohto Pharmaceutical Co., Ltd. Ginger extract powder at doses of 25 mg or 50 mg was administered in each group. This experiment included 5 eyes of 4 rats in a 25-mg intake group, and 7 eyes of 4 rats in a 50-mg intake group. First, all rats underwent LSFG measurement at baseline. One week later, the rats were given either 25 mg or 50 mg of ginger extract powder sublingually, anesthetized after 10 min, and then placed on a heat pad. After another 30 min, LSFG measurements were taken in the same eyes as those imaged at baseline. To minimize measurement bias, we alternated measurements among the rats from each group. The post-intake MBR was compared with the baseline MBR.

### Intravitreal injection of ET-1 in rats as an experimental model of ischemia

Two microliters of ET-1 were injected at 1.25, 2.50, or 5.00 pmol. The vehicle (PBS) group consisted of 7 rats. The 1.25 pmol, 2.50 pmol, and 5.00 pmol groups had 5, 16, and 7 rats, respectively. All intravitreal injections of ET-1 were performed in the right eye. The injection was performed as follows: the rats were anesthetized, and 10 min later, an intravitreal injection of either ET-1 or, in the controls, PBS was performed. All injections had an equal volume and method of administration. After intravitreal injection, the rats were placed on a heat pad, and after 20 min, LSFG measurement was performed. Comparisons across all groups were consistently carried out on the same day. Measurements were rotated among the rats across the different groups to reduce the potential for measurement bias.

### Experiments on ginger extract intake in the rat model of ischemia

In this experiment, three groups were included. (1) The sham group consisted of 7 rats that received an intravitreal injection of 2 μl of PBS and did not receive any ginger extract. (2) The ginger extract intake group, which comprised 11 rats, was first given a sublingual dose of 50 mg of ginger extract powder. After 10 min, they were anesthetized and, an additional 10 min later, received an intravitreous injection of 2 μl of ET-1 at a concentration of 2.50 pmol. LSFG measurement was conducted 20 min after ET-1 injection. (3) The control (non-intake) group, consisting of 12 rats, received the identical intravitreal injection of ET-1 as the ginger extract intake group but did not receive any ginger extract.

For each animal, two different investigators were involved as follows: a first investigator (NM) administered the ginger extract and carried out the anesthesia. A second investigator (NT) performed the ET-1 injection and LSFG measurement in a blinded manner, unaware of whether the ginger extract had been administered or not. For each group, comparisons were made on the same day. To ensure minimal measurement bias, we varied the order of measurements among the rats in each group.

### Statistical analysis

Data within the tables and the text are expressed as the mean ± SD. For each boxplot, the central line indicates the median of the dataset. The top and bottom boundaries of the box represent the third and first quartiles respectively. The whiskers extend to the maximum or minimum data value. The percentage change in MBR compared to baseline was calculated. A linear mixed-effects model was used to determine the statistical difference in %MBR in each phase of the ginger extract powder dose experiments, setting the “subject variable” as a random effect. ANOVA was used to analyze the significance of differences in demographic characteristics in the ET-1 intravitreal injection study and the ginger extract intake study. The Tukey–Kramer test was used to analyze the significance of differences in %MBR values in the ET-1 intravitreal injection study and the ginger extract intake study. All statistical analyses were performed with R software (version 3.2.5) or JMP software (Pro version 16.1.0; SAS Institute Japan, Inc., Tokyo, Japan). The significance level was set at *P* < 0.05.

### Ethical approval

This study adhered to the ARVO Statement Guidelines for the Use of Animals in Ophthalmic and Vision Research. All experimental protocols were approved by the Committee for Animal Experiments at the Tohoku University Graduate School of Medicine (Approval #2022-056).

## Results

### Ginger extract powder dosage

**Figure 1:**
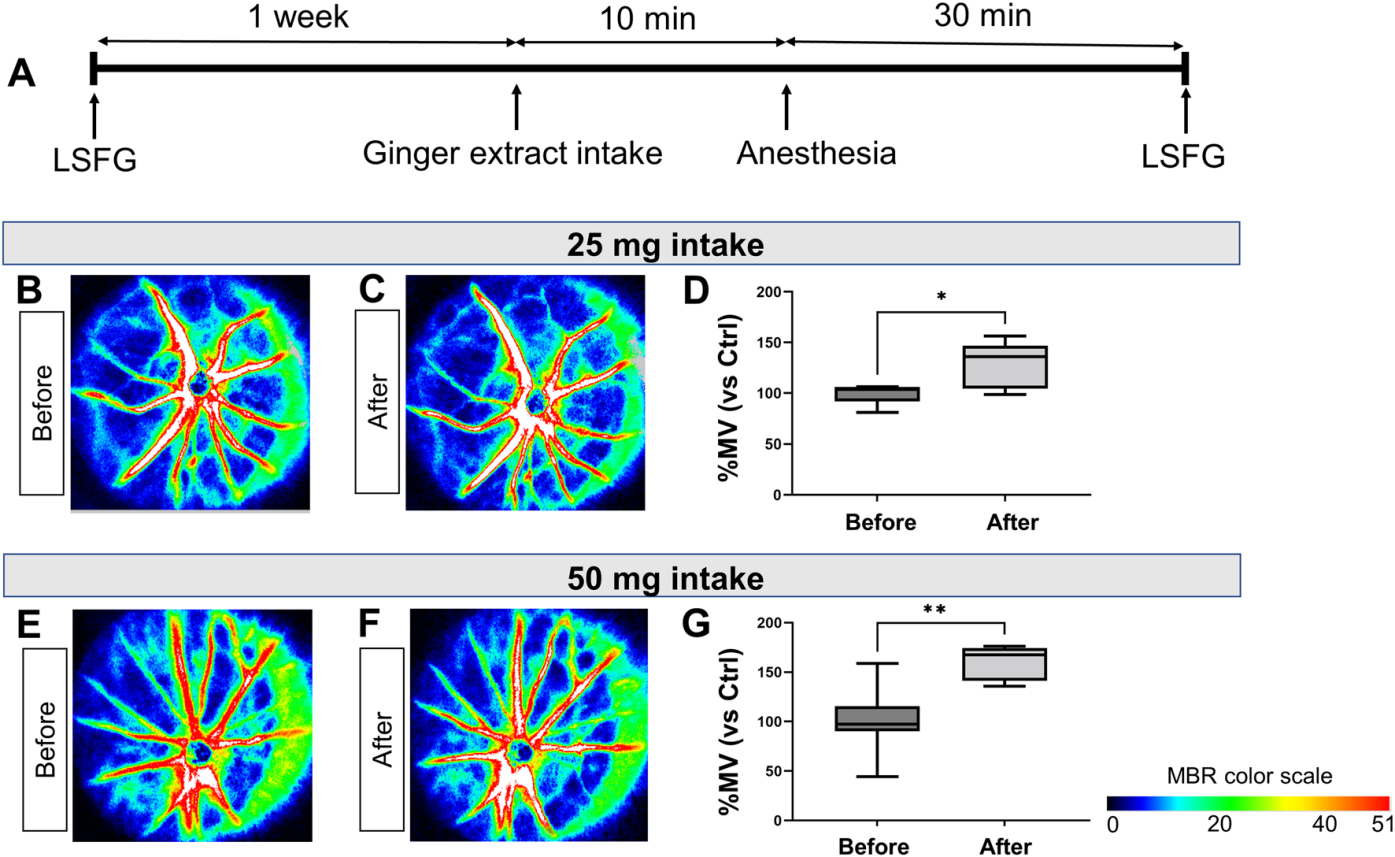
Administration of a ginger extract powder increased optic nerve head blood flow (ONH BF) in normal rats. (**A**) Time course of the experimental design, including the timeline for intake, anesthesia, and measurements of vessel-area mean blur rate (MV). (**B**, **C**) Representative color map images of ONH BF before and after intake of 25 mg of ginger extract and (**D**) quantitative box plot of %MV (n = 5). (**E**, **F**) Representative color map images of ONH BF before and after intake of 50 mg of ginger extract and (**G**) quantitative box plot of %MV (n = 7). *P < 0.05, **P < 0.01 in a linear mixed-effects model.

First, we investigated the effects of the ginger extract on ONH BF in normal rats. The experimental design is shown in Fig. 1A. Figure 1B and C represent the same subject before and after, respectively, the administration of 25 mg of ginger extract, while Fig. 1D shows a box plot of the quantitative results. Similarly, Fig. 1E and F represent the same subject before and after the administration of 50 mg of ginger extract, while Fig. 1G shows a box plot of the quantitative results. In the ginger extract powder intake experiment, the percentage changes in MBR after intake compared to baseline were as follows: in the 25 mg group, %MV was 127.7 ± 23.1% (Fig. 1D). In the 50 mg group, %MV was 161.0 ± 16.1% (Fig. 1G). In both groups, %MV post-intake was significantly higher than at baseline (*P* = 0.042, *P* = 0.001, respectively), with the increase being more pronounced in the 50 mg group. %MT and %MA showed similar trends to %MV (Supplementary Table 1).

### ET-1 intravitreal injection in the rat model of ischemia

**Figure 2:**
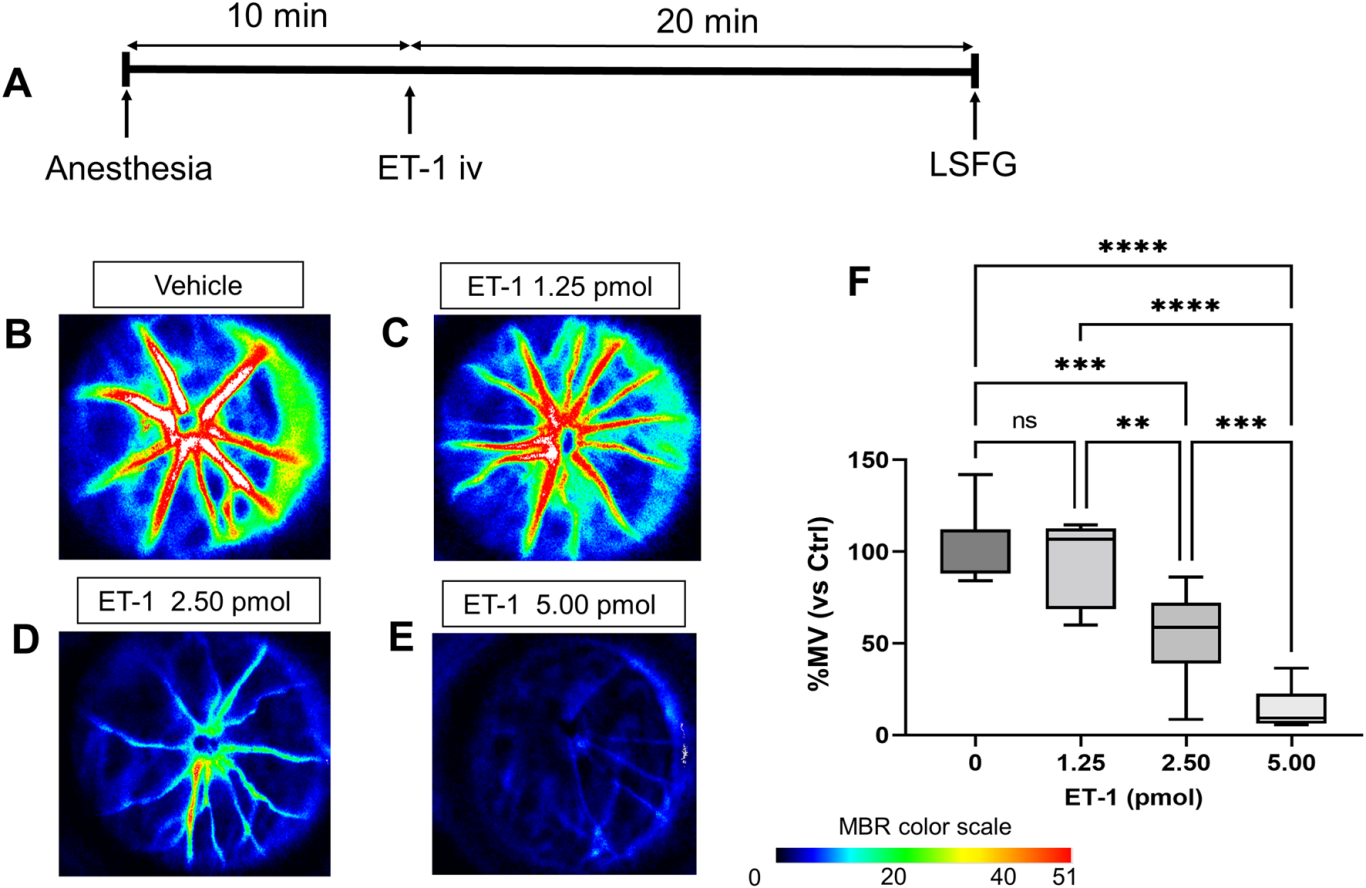
Change in optic nerve head blood flow (ONH BF) of rats following ET-1 intravitreal injection (iv). (**A**) Time course of the experimental design showing the timeline for induction of anesthesia, ET-1 intravitreal injection, and measurements of vessel-area mean blur rate (MV). Representative color maps of ONH BF in rats that received an injection of (**B**) phosphate-buffered saline, (**C**) 1.25 pmol ET-1, (**D**) 2.50 pmol ET-1, and (**E**) 5.00 pmol ET-1. (**F**) Box plot showing the quantitative measurement of %MV 20 min after the intravitreal injection of ET-1 and PBS (n = 7), 1.25 pmol ET-1 (n = 5), 2.50 pmol ET-1 (n = 16), and 5.00 pmol ET-1 (n = 7). **P < 0.01, ***P < 0.001, ****P < 0.0001 in a one-way ANOVA followed by the Tukey–Kramer test.

Next, we evaluated ET-1-induced ONH BF reduction, because to the best of our knowledge, there has been no previous study comparing the degree of ischemia in the ONH of rats after the injection of ET-1 at different concentrations. The experimental design is shown in Fig. 2A. Representative LSFG-Micro color map images with or without ET-1 injection are shown in Figs. 2B–E. The percentage changes in MBR compared to controls were as follows. In the 1.25 pmol group, %MV was 93.9 ± 24.0%. In the 2.50 pmol group, %MV was 54.5 ± 21.2%. In the 5.00 pmol group, %MV was 14.6 ± 11.3%. The 1.25 pmol group showed no significant differences in %MV, %MT, or %MA (*P* = 0.953, *P* = 0.647, and *P* = 0.910, respectively). The 2.50 pmol group and the 5.00 pmol group showed a significant concentration-dependent decrease in ONH BF compared to the controls (%MV: *P* = 0.0001, *P* < 0.0001; %MT:* P* = 0.0006, *P* < 0.0001; %MA: *P* < 0.0001, *P* < 0.0001, respectively, Fig. 2F). The measurement characteristics of the ET-1 intravitreal injection subjects are shown in Table 1. Besides LSFG parameters, there were no significant differences in IOP, MBP, SBP, DBP, MOPP, or HR between the groups (*P* = 0.095 − 0.726).

**Table 1:** Demographic characteristics of rats following intravitreal injection of ET-1.

Variables	Vehicle	ET-1 groups			*P* value
		1.25 pmol/rat	2.50 pmol/rat	5.00 pmol/rat	
Number of rats, n	7	5	16	7	–
Intraocular pressure, mmHg	6.2 ± 0.7	6.3 ± 0.8	5.4 ± 0.5	5.3 ± 0.7	0.600
Mean BP, mmHg	79.2 ± 4.9	86.4 ± 5.8	84.4 ± 3.6	86.7 ± 4.9	0.707
Systolic BP, mmHg	91.4 ± 6.0	100.5 ± 7.1	100.1 ± 4.4	100.3 ± 6.0	0.639
Diastolic BP, mmHg	73.1 ± 4.4	78.8 ± 5.3	76.6 ± 3.3	79.9 ± 4.4	0.726
Ocular perfusion pressure, mmHg	46.6 ± 3.3	51.1 ± 3.9	50.9 ± 2.4	52.5 ± 3.3	0.625
Heart rate, bpm	232.1 ± 22.9	255.2 ± 40.5	257.5 ± 15.4	236.6 ± 25.1	0.095
% Vessel-area MBR, % (Vessel-area MBR, AU)	100.0 ± 20.7 (68.1 ± 5.1)	93.9 ± 24.0 (63.9 ± 6.1)	54.5 ± 21.2 (37.1 ± 3.4)	14.6 ± 11.3 (10.0 ± 5.1)	< 0.0001*
% Tissue-area MBR, % (Tissue-area MBR, AU)	100.0 ± 43.5 (16.5 ± 7.2)	83.4 ± 19.1 (13.7 ± 3.1)	51.4 ± 16.7 (8.5 ± 2.7)	22.5 ± 13.5 (3.7 ± 2.2)	< 0.0001*
% Overall-area MBR, % (Overall-area MBR, AU)	100.0 ± 12.2 (48.8 ± 6.0)	93.2 ± 27.9 (45.5 ± 13.6)	41.0 ± 18.4 (20.0 ± 9.0)	11.7 ± 7.9 (5.70 ± 3.9)	< 0.0001*

ANOVA was used for group comparison.

*BP* Blood pressure; *bpm* Beats per minute; *AU* Arbitrary unit; *MBR* Mean blur rate.

*indicate the statistical significance.

### Ginger extract intake in a rat model of ischemia

**Figure 3:**
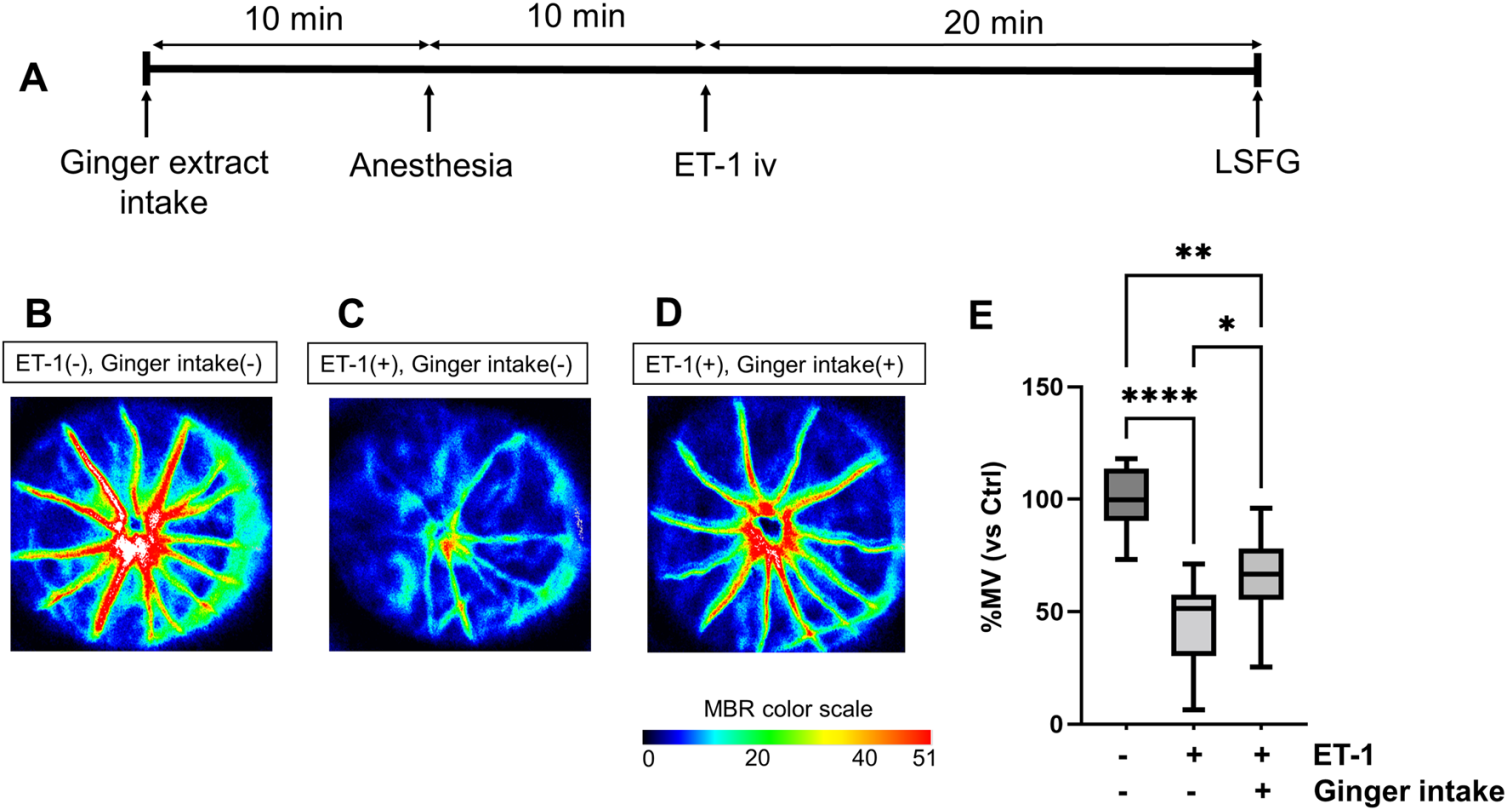
Administration of the ginger extract powder ameliorated the reduction of optic nerve head blood flow (ONH BF) in rats that received ET-1 intravitreal injection (iv). (**A**) Time course of the experimental design, showing the timeline for intake, anesthesia, ET-1 intravitreal injection, and measurements of vessel-area mean blur rate (MV). (**A**–**C**) Representative color maps of ONH BF in rats that received (**B**) a phosphate-buffered saline (PBS) iv (**C**), an ET-1 iv, and (**D**) intake of the ginger extract and ET-1 iv. (**E**) Box plot showing the change in %MV 20 min after the PBS iv (n = 7), ET-1 injection (n = 12), and intake of the ginger extract and ET-1 iv (n = 11). *P < 0.05, **P < 0.01, ****P < 0.0001 in a one-way ANOVA followed by the Tukey–Kramer test.

Finally, we evaluated whether the ginger extract powder could improve ONH BF under ET-1-induced ONH BF impairment. In this experiment, we selected the rats that received ET-1 at a concentration of 2.50 pmol, because ONH BF began to significantly decrease at an ET-1 concentration of 2.50 pmol in the previous experiment. The experimental design is shown in Fig. 3A. Representative LSFG-Micro color maps image of each group are shown in Fig. 3B–D.

The percentage changes in MBR in the case group compared to the sham (PBS injection and non-intake) group were as follows. In the case (ET-1 injection and ginger extract intake) group, %MV was 64.7 ± 19.2%. In the control (ET-1 injection and non-intake) group, %MV was 44.0 ± 20.6%. %MV and %MT were significantly higher in the ginger extract intake group than the non-ginger extract intake group (*P* = 0.039, *P* = 0.046, respectively, Fig. 3E), indicating that the ginger extract ameliorated the dysregulation of ONH BF. Similarly, %MA in the ginger extract intake group tended to be higher than the non-ginger extract intake group (*P* = 0.068). On the other hand, there were no significant differences in IOP, MBP, SBP, DBP, MOPP, or HR between the case (intake) group and the control (non-intake) group (*P* = 0.069 − 0. 695) (Table 2).

**Table 2:** Demographic characteristics of rat ischemia models with ginger extract intake.

Variables	ET-1 iv (+), ginger intake (−)	ET-1 iv (+), ginger intake (+)	*P* value
Number of rats, n	11	12	–
Intraocular pressure, mmHg	4.8 ± 0.7	5.0 ± 0.3	0.635
Mean BP, mmHg	73.0 ± 7.2	68.3 ± 6.1	0.103
Systolic BP, mmHg	85.3 ± 2.3	79.4 ± 2.4	0.088
Diastolic BP, mmHg	66.9 ± 1.9	62.8 ± 1.9	0.137
Ocular perfusion pressure, mmHg	43.9 ± 4.3	40.6 ± 4.0	0.069
Heart rate, bpm	253.4 ± 21.2	263.1 ± 19.6	0.267
% Vessel-area MBR, % (Vessel-area MBR, AU)	44.0 ± 20.6 (25.6 ± 12.0)	64.7 ± 19.2 (37.7 ± 11.2)	0.022*
% Tissue-area MBR, % (Tissue-area MBR, AU)	42.3 ± 18.8 (7.0 ± 3.1)	59.7 ± 16.1 (9.9 ± 2.7)	0.027*
% Overall-area MBR, % (Overall-area MBR, AU)	33.5 ± 18.9 (14.3 ± 8.0)	52.7 ± 18.3 (22.4 ± 7.8)	0.022*

ANOVA was used for group comparison.

*BP* Blood pressure; *bpm* Beats per minute; *AU* Arbitrary unit; *MBR* Mean blur rate.

*indicate the statistical significance.

## Discussion

High IOP is the only modifiable risk factor for the onset and progression of glaucoma, but the existence of normal-tension glaucoma (NTG) indicates that it is not the only risk factor. Chronic BF dysfunction in the ONH is also a risk factor for glaucoma, and this phenomenon may suggest possible new interventions. In the current study, we found that a ginger extract improved ONH BF in rats that received an intravitreal injection of ET-1. This suggests that this ginger extract may be able to prevent BF dysfunction.

Dysregulation of ONH BF has been suggested to be associated with NTG^9^. Flammer et al. have noted the presence of migraine cold extremities in patients with NTG and have mentioned potential contribution of excessive peripheral vasoconstriction due to endothelial dysfunction to the pathogenesis of NTG. They refer to this phenotype as Flammer syndrome^24–26^. Improving ONH BF by dilating these excessively constricted vessels may have therapeutic potential for glaucoma. Our previous study showed that intake of kampo medicines (traditional Japanese medical formulas based on plants) increased ONH BF in healthy subjects and patients presenting with typical symptoms of Flammer syndrome^27,28^. We have also previously reported that the topical administration of tafluprost, a type of FP agonist, significantly increased ONH BF in the eyes of normal subjects and patients with NTG^29^. Furthermore, we found that a drug delivery system that included tafluprost prevented RGC death in rats independently of IOP^30^. These findings suggest that improving ONH BF may have the potential to attenuate the loss of RGCs. Given this, ginger extract supplement might harbor therapeutic potential for glaucoma by improving ONH BF.

ET-1 is a potent vasoconstriction factor^10,31^, and the level of ET-1 in the plasma has been reported to be significantly elevated in NTG patients^32–38^. Many animal experiments have reported that the intravitreal injection of ET-1 induces optic neuropathy and RGC loss^11–13^. However, to our knowledge, there have been no reports measuring the reduction in ONH BF after the intravitreal administration of ET-1. The current study is thus the first to report on the administration of different concentrations of ET-1 and to measure changes in ONH BF using LSFG, a method that quantifies MBR and is highly correlated with hydrogen clearance method^39^. LSFG is noninvasive and has become increasingly popular in recent years in clinical use. Therefore, this study’s measurements of the effect of ET-1 on ONH BF provided valuable data that may be applicable to future clinical studies.

The ginger extract we used in this study contained [6]-shogaol, which is known in kampo medicine. A previous study showed that [6]-shogaol increased intestinal blood flow (IBF), and that this effect was mediated by the calcitonin gene-related peptide (CGRP) and its receptor^18^. Murata et al.^40^ reported that [6]-shogaol increased IBF in a dose-dependent manner in rats. This effect of [6]-shogaol on IBF hyperemia is mainly mediated by CGRP, which subsequently leads to the activation of endothelial nitric oxide synthase. Our supplement, which contains [6]-shogaol, improved ONH BF less than 30 min after administration, which is a similar timeframe as was observed in the IBF study^18^. This suggests that [6]-shogaol is an active component in our supplement and may be useful in the treatment of diseases associated with vascular dysregulation, such as NTG.

The present study has several limitations. Firstly, we were unable to empirically verify whether the increase in ONH BF leads to neuroprotective effects in this study. The relationship between improved ONH BF and neuroprotection warrants further investigation. Secondly, we did not evaluate the duration for which the increased MV maintained. We tracked alterations for only 40 min, constrained by the anesthetic's 40-min limitation.

In conclusion, the present study demonstrates that a ginger extract containing [6]-shogaol has the potential to increase ONH BF under conditions of both good health and ischemia independently of BP and IOP. Thus, our ginger extract containing [6]-shogaol may become a useful treatment for NTG and help prevent optic neuropathy induced by vascular dysregulation, such as occurs in NTG.

### Supplementary Information

Supplementary Figure S1.

Supplementary Table S1.

## Data Availability

The datasets used and/or analyzed during the current study are available from the corresponding author on reasonable request.
