# Supplementary figures and images for: A ginger extract improves ocular blood flow in rats with endothelin-induced retinal blood flow dysfunction
Source: Sci Rep. 2023 Dec 20;13:22715. doi: 10.1038/s41598-023-49598-w (PMC10733345; doi:10.1038/s41598-023-49598-w)

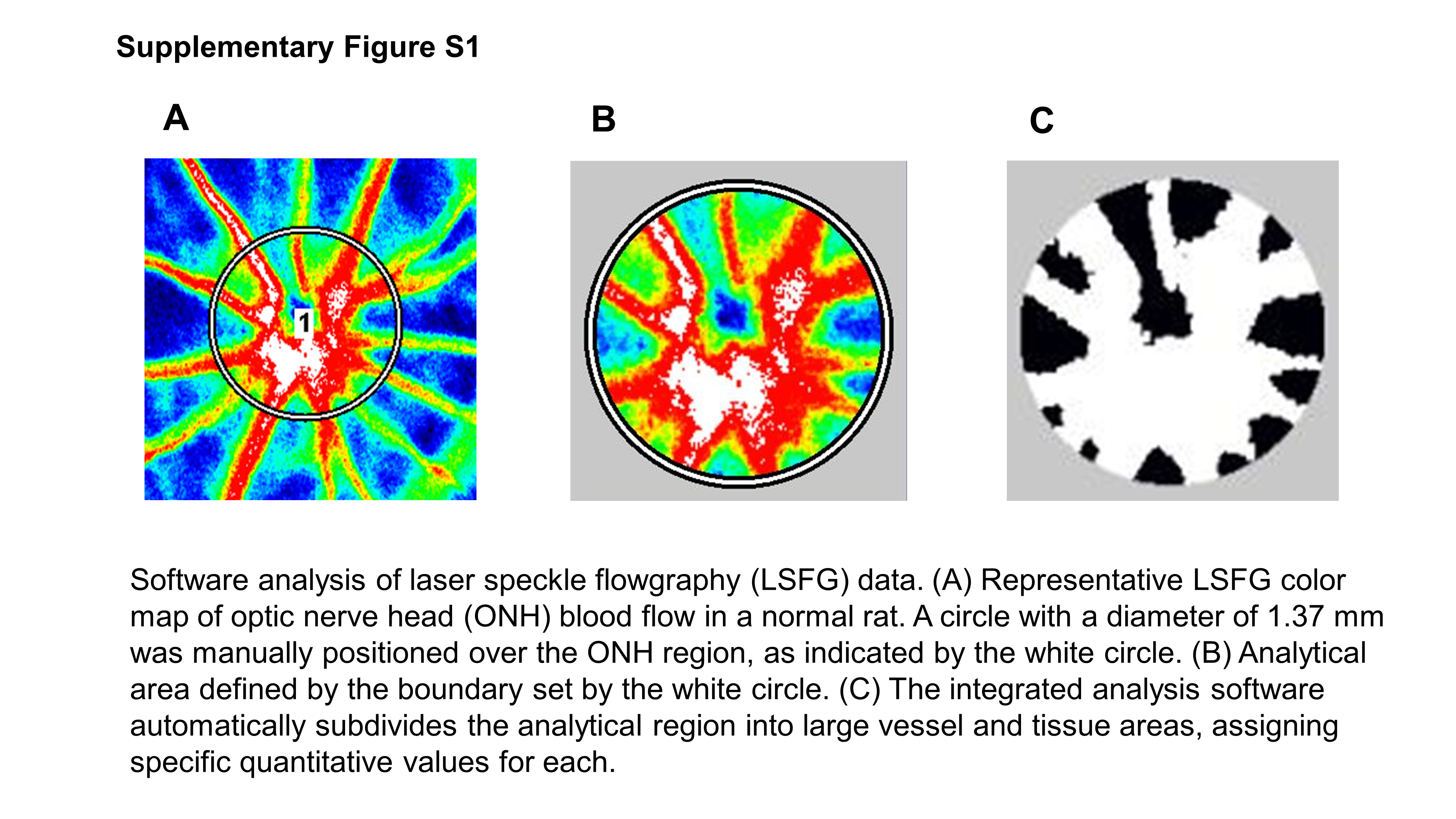

Supplement: Supplementary file 1 — Supplementary Figure S1. [file 41598_2023_49598_MOESM1_ESM.tif]
