# Supplementary material for: A ginger extract improves ocular blood flow in rats with endothelin-induced retinal blood flow dysfunction
Source: Sci Rep. 2023 Dec 20;13:22715. doi: 10.1038/s41598-023-49598-w (PMC10733345; doi:10.1038/s41598-023-49598-w)
Supplement: Supplementary file 2 — Supplementary Table S1. [file 41598_2023_49598_MOESM2_ESM.docx]

| Supplementary Table 1. Comparison of LSFG parameters before and after ginger extract administration | | | | | |
| --- | --- | --- | --- | --- | --- |
|  | | | | | |
| Variables | |  | Before | After | *P* value |
| % Vessel-area MBR, %  Vessel-area MBR, AU | 25mg |  | 100.0 ± 10.8  （48.6 ± 4.7） | 127.7 ± 23.1  （62.0 ± 10.0） | 0.042* |
|  | 50mg |  | 100.0 ± 34.0  （41.4 ± 13.1） | 161.0 ± 16.1  （66.7 ± 6.2） | 0.001* |
| % Tissue-area MBR, %  Tissue-area MBR, AU | 25mg |  | 100.0 ± 22.2  （12.4 ± 2.8） | 126.5 ± 16.3  （15.7 ± 2.0） | 0.064 |
|  | 50mg |  | 100.0 ± 19.8  （12.5 ± 2.5） | 115.5 ± 22.2  （14.5 ± 2.8） | 0.192 |
| % Overall MBR, %  Overall MBR, AU | 25mg |  | 100.0 ± 15.1  （36.3 ± 5.5） | 113.9 ± 21.0  （41.4 ± 7.6） | 0.155 |
|  | 50mg |  | 100.0 ± 21.7  （34.0 ± 7.4） | 138.7 ± 23.6  （47.1 ± 8.0） | 0.008* |
|  |  |  |  |  |  |
| MBR = mean blur rate. |  |  |  |  |  |
| *P* value: linear mixed-effects model. *statistical significance. | | | | | |
